# Specific effects of Pulsed Field Ablation on the cardiac tissue, the nervous system and the conduction system in experimental and clinical studies

**DOI:** 10.1093/europace/euag208

**Published:** 2026-10-21

**Authors:** Josselin Duchateau, Iwanari Kawamura, Gurukripa N Kowlgi, Elad Maor, Moritz Nies, Christian W Zemlin, Tomás García-Sánchez, Samuel J Asirvatham

**Affiliations:** Hôpital Cardiologique Haut-Lévêque, CHU de Bordeaux, L'Institut de RYthmologie et modélisation Cardiaque (LIRYC), Université de Bordeaux, Passac Cedex, France; Department of Cardiovascular Medicine, Institute of Science Tokyo, Tokyo, Japan; Department of Cardiovascular Diseases, Mayo Clinic, Rochester, MN, USA; Division of Cardiology, Sheba Medical Center, Ramat-Gan, Israel; Tel Aviv University Gray School of Medicine, Tel-Aviv, Israel; Department of Cardiology, University Medical Center Hamburg-Eppendorf, Hamburg, Germany; Division of Cardiothoracic Surgery, Washington University School of Medicine, Barnes-Jewish Hospital, St. Louis, MO, USA; Department of Biomedical Engineering, Washington University in St. Louis, St. Louis, MO, USA; Department of Medical Education, Boston Scientific Corporation, Madrid, Spain; Department of Cardiovascular Diseases, Mayo Clinic, Rochester, MN, USA

**Keywords:** Pulsed field ablation, Preferential myocardial ablation, Tissue selectivity, Lesion formation, Heterogeneous substrate, Phrenic nerve, Vagal responses, Conduction system

## Abstract

**Aims:**

Pulsed field ablation (PFA) has emerged as a novel ablation modality for the treatment of cardiac arrhythmias and has rapidly reshaped contemporary electrophysiology practice. This article provides a comprehensive analysis on the mechanisms underlying the specific effects of PFA on myocardial, neural tissue structures, and the cardiac conduction system, integrating biophysical principles with preclinical and clinical evidence relevant to contemporary electrophysiology practice.

**Methods and results:**

This document provides an exhaustive mechanistic explanation for the preferential effects of PFA on myocardium and the limits of the ‘tissue-selectivity’ concept. The complex interplay between cellular and tissue-level biophysics, biological responses, and tissue regenerative capacity that determines differential susceptibility to electroporation across tissues is analysed. Distinct patterns of lesion formation and maturation following PFA are also reviewed, highlighting the temporal and histopathological differences compared with radiofrequency ablation. Additionally, lesion formation in heterogeneous substrates is analysed, including fibrotic and adipose-rich myocardium, where local electric field distribution critically influences lesion durability.

**Conclusion:**

The article further examines the interaction between pulsed electric fields and neural tissue, with emphasis on the phrenic nerve and intrinsic cardiac autonomic nervous system. While nerve stimulation and transient reversible conduction disturbances produced by reversible electroporation can occur during ablation, permanent neural injury appears extremely rare in current clinical experience. Finally, this manuscript examines current understanding regarding the specific effects of PFA on the cardiac conduction system, which according to the limited available evidence appear strongly dependent on electric field dose and anatomical proximity.

## Graphical Abstract

**Graphical Abstract:**
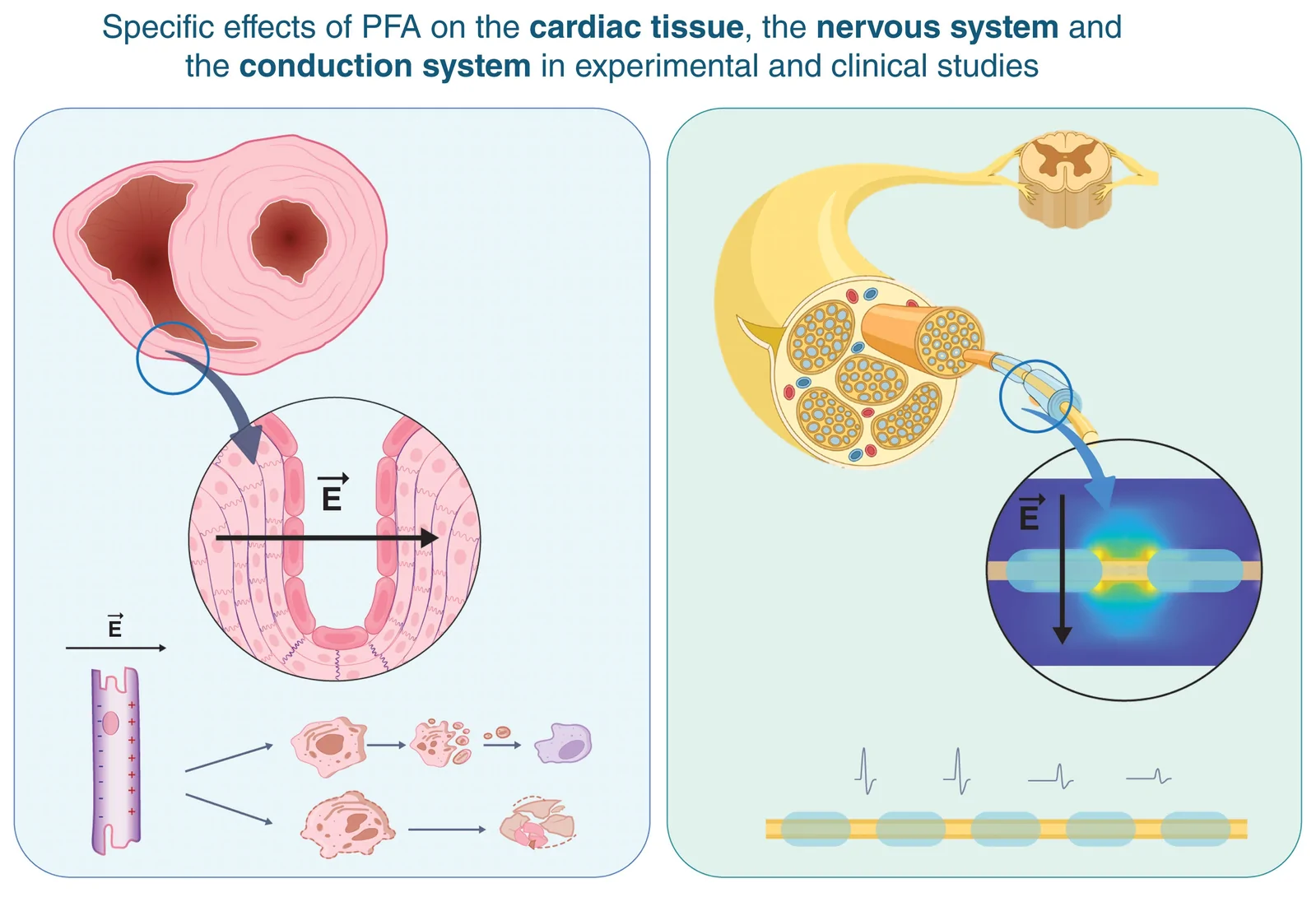

## Introduction

The term pulsed field ablation (PFA) was first used in a clinical publication in 2019 to refer to the specific use of irreversible electroporation (IRE) for the ablation of myocardial tissue in the treatment of cardiac arrhythmias.^1^ Electroporation is a general phenomenon that describes the increase in cell membrane permeability leading to reversible or irreversible effects depending on the electric dosing. It is a broad biophysical phenomenon that can be observed in every cell type and even in artificial lipid bilayers. In this article, we will focus on the theoretical arguments and experimental evidence that can explain the specific responses of cardiac and nerve tissues to electroporation both from a biophysical and a biological perspective and its implications for clinical practice.

It is important to emphasize that the purpose of this review is to summarize common observations and underlying mechanisms associated with PFA. Nevertheless, caution should be exercised when generalizing these concepts across different PFA technologies, as important differences may exist between platforms.^2^ Variations in waveform design, energy delivery, and electrode configuration may influence both the extent and nature of tissue responses. For instance, although PFA is widely considered a non-thermal ablation modality because electroporation is the predominant mechanism of cell injury, temperature increases will always exist due to Joule heating. Consequently, thermal contributions to tissue injury may vary between platforms, potentially leading to differences in the biological response when thermal exposure is not adequately controlled or minimized.

The first part of the article will focus on the specific properties of cardiac tissue that contribute to its high sensitivity to electroporation. The second part of this article will focus on the effects of electroporation on nerves with a specific focus on neural structures that are relevant for cardiac ablation procedures.

## Part I: PFA effects on cardiac tissue

### Preferential myocardial ablation

#### Revisiting the selectivity concept

When the first modern PFA systems were introduced for treating cardiac arrhythmias, PFA was described as a tissue-selective ablation modality.^1,3^ This term was used to describe the potential of PFA to spare collateral structures as the oesophagus or the phrenic nerve during pulmonary vein isolation (PVI) procedures and is one of the main advantages of this ablation modality.

While the ‘selectivity’ concept is still widely used by part of the EP community, a more scientifically accurate description of PFA is that it is a *preferential* myocardial ablation modality.^4,5^ While this change of wording seems subtle, it proves how the growing scientific evidence about PFA is helping evolve our understanding about this ablation modality. Additionally, this shift in terminology may also have important implications in refining the perception of clinical users about PFA safety limits and optimization of their clinical practice. The initial selectivity concept could lead to the misconception that PFA only affects cardiomyocytes, without any associated risks to other tissue types. Nowadays, the scientific community widely accepts that while PFA displays a wider safety margin than thermal ablation modalities, side effects are still possible in clinical practice.

Both the selectivity concept and the updated preferential ablation concept were born from scientific evidence and experimental observations. Tissue-specific effects were initially reported in many studies using IRE for tumour ablation.^6–8^ Subsequently, similar findings were observed in the cardiac arrhythmia ablation space. Detailed chronic histological studies in preclinical models systematically observed preferential fibrotic replacement of cardiomyocytes with preservation of other intramyocardial or surrounding structures such as nerves or vasculature.^5,9–12^

Following this, we will review the main concepts related to the preferential ablation properties of PFA and will discuss the current limits and knowledge gaps about this phenomenon.

#### Experimental evidence for preferential ablation

Experimental evidence accumulated not only in cardiac ablation applications but also in the field of tumour ablation with IRE, has repeatedly demonstrated, both *in vitro* and *in vivo*, specific effects and differential cell death susceptibility of different cells or tissues.^5,13,14^ While specific *in vivo* comparative studies for cardiac tissue are still scarce, some works have shown that myocardial tissue was more susceptible to IRE than vascular, neural, hepatic, and skeletal muscle tissues^5^ and have provided experimental evidence for higher susceptibility of cardiac tissues compared to other tissues except for the skeletal muscle.^15^ However, the waveform parameters used in these studies significantly differ from the short biphasic pulses generally used in PFA commercial applications.

From a simplified perspective, the preferential effects of PFA on cardiomyocytes have been attributed to a significantly lower electric field electroporation threshold than other tissues, rendering cardiac cells more susceptible to the electric field exposure than other cell types. An important consideration is to understand that the electric field threshold value depends on the PFA-specific waveform and other parameters such as the duration of field exposure, its distribution over time or the repetitive dosing strategy.^15–17^ This concept has important implications when trying to compare different tissue types or even different PFA systems because threshold is system-specific and there is not a unique value that can be generalized.

Some initial studies tried to provide proof of a lower cardiomyocyte threshold extracting data from very different studies with very different waveforms and experimental conditions, limiting the value of these initial comparisons.^1^ More recently, *in vitro* studies were able to demonstrate a lower electric field threshold for cardiomyocytes compared to smooth muscle cells and neurons under identical experimental conditions.^5,13,14^

In the following section, we will focus on the characteristics of cardiomyocytes and tissue that affect their susceptibility to IRE.

#### Biophysical properties of cardiomyocytes

It has been extensively experimentally and theoretically studied that the necessary condition for initiating electroporation is reaching a critical transmembrane voltage.^18^ From a purely biophysical perspective, the stationary transmembrane voltage induced by an external field varies in magnitude and direction across the cell membrane, depending on the orientation of the membrane elements relative to the field. The maximum induced voltage across the whole membrane of a cell is proportional to cell size, and for non-spherical cells, the eccentricity can either amplify or reduce the maximum induced transmembrane voltage, depending on the cell’s orientation.^19,20^ Thus, the electroporation threshold (pore formation probability) has been shown to correlate to some extent to biophysical magnitudes such as cell radius, geometry, orientation, and cytoplasm/nucleus ration.^21–23^

Biophysics predicts that, for electroporation using pulses in the microsecond-duration range, larger cells will be more susceptible than small cells. Cardiomyocytes are large, elongated cells, often exceeding 100 µm in length. In this way, the geometry of individual cardiomyocytes makes them particularly sensitive to electroporation (see *Figure 1*). However, it is important to consider that the characteristics of the PFA waveform, specifically, pulse duration, will strongly affect the impact of biophysical parameters (size and orientation). For example, experimental *in vitro* evidence demonstrated that dependence on orientation is much less relevant when using biphasic, short microsecond-duration waveforms compared to longer pulses.^24^

**Figure 1:**
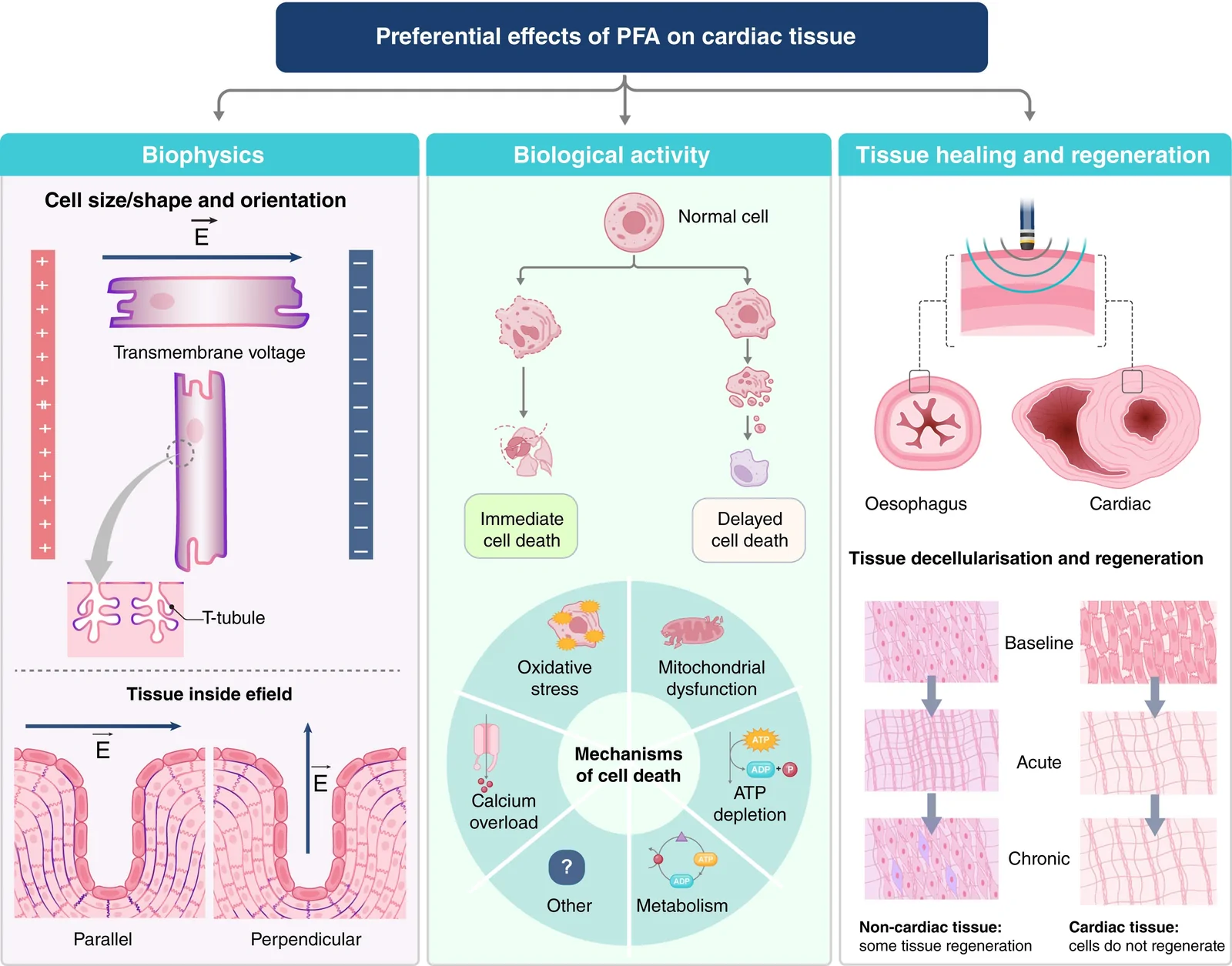
Summary of preferential effects of PFA on cardiac tissue. (*A*) Schematic representation of the transmembrane voltage induced under an electric field in different directions at the cell and tissue levels. (*B*) Summary of main biological mechanisms involved in cardiomyocyte cell death after PFA. (*C*) Example of differential response of cardiac tissue and oesophageal tissue in the event of acute damage by PFA; as extracellular matrix is preserved, oesophageal cells have the ability to regenerate and tissue heals, cardiac tissue does not have the ability to regenerate.

Cardiomyocytes also possess an extensive transverse-tubule system—an invaginated continuation of the sarcolemma that greatly expands membrane surface area and tightly coupled membrane integrity to calcium handling machinery. Severe electroporation therefore may disrupt not only the outer membrane but also a complex internal membrane network, facilitating increased cell death.^3^

Cellular biophysics cannot fully explain PFA effects in actual cardiac tissue because the electric field distribution in tissue is strongly affected by its architecture, including cell-to-cell coupling, the presence of electrically insulating structures, distribution of cell types with different electrical properties, and tissue anisotropy.^25^

#### Biophysical properties of cardiac tissue

Cardiac tissue is highly cellular and electrically conductive, with relatively little insulating extracellular matrix in non-fibrotic atrial myocardium. This favours penetration of electric fields rather than dissipation through collagen-rich stroma. In contrast, other tissues have a more complex architecture that can greatly impact electric field distribution. Peripheral nerves are bundled within multilayered connective tissue sheaths and frequently insulated by myelin, while vessels and hollow organs contain layered smooth muscle and elastic components that may impact effective electric field distribution and thus their susceptibility to electroporation.^26–29^

Additionally, cardiomyocytes form a functional syncytium via abundant gap junction coupling at intercalated discs, enabling low-resistance current flow between neighbouring cells. As a result, the classical single-cell intuition that induced transmembrane voltage scales primarily with cell size and orientation as discussed above, is only partially applicable to intact heart tissue, where tissue geometry, conductivity anisotropy, and intercellular coupling shape the field gradients that drive electroporation. Myocardial tissue architecture exhibits pronounced electrical and structural anisotropy arising from the aligned arrangement of elongated cardiomyocytes into fibre bundles and laminar sheets (see *Figure 1*). At the macroscopic scale, this produces a conductivity tensor with substantially higher conductivity along the local fibre axis than transverse to it.^30–32^ During PFA, the imposed quasi-static electric field, therefore, redistributes through the myocardium in a fibre-orientation–dependent manner with the electric field expected to distribute preferentially along the fibres leading to non-symmetrical lesions.^33^ However, recent computational models have demonstrated that lesion shape observed with focal PFA catheters would fit to the electric field distribution at a macroscopic level alone but to more complex predictions based on relative cell membrane damage at a microscopic level.^30^

The high content of cardiomyocytes promotes preferential ablation. The fact that cardiac tissue forms a syncytium does not necessarily increase or decrease PFA efficacy, but it profoundly affects the field distribution in cardiac tissue and means that PFA efficacy is ultimately an emergent property of the coupled anisotropic tissue network: local electroporation probability reflects not only cardiomyocyte membrane biophysics but also the structure-dependent redistribution of the electric field within the syncytium.

Besides the biophysical properties that help to explain how electric fields damage cell membranes, the ultimate fate of electroporated cells and tissues also depends on their biology, specifically on their ability to repair membranes, maintain homeostasis and replace damaged cells.^34^

#### Biological properties of cardiomyocytes

Cell biology must be considered as an important element in the complex interplay between electroporation and resulting cell death. In vitro studies have provided some general biological mechanisms for cell death induced by electroporation that could explain why cardiac tissue could undergo preferential ablation with PFA (see *Figure 1*).^34^ Among them, one of the most relevant is the differential ability for cell membrane repair after electroporation. This was observed between normal and cancer cells in a process mediated by calcium-dependent mechanisms.^35–37^ It was also shown in human-induced pluripotent stem cells, where regulated cell death played an important role following PFA.^38^ However, other factors such as cytoskeleton integrity could also be important.^39^ Calcium homeostasis could also play a crucial role in cell death after electroporation, leading to mitochondrial dysfunction and ATP depletion.^40^ Differences in mitochondrial properties, internal calcium reserves, etc., between different cell types could also contribute to the preferential cell death observed in cardiomyocytes.

In fact, electroporation-related disruption associated with Ca^2+^ influx and mitochondrial dysfunction has been described as a key pathway leading from severe membrane injury to cell death in excitable cells, including cardiomyocytes.^34,38,41,42^ As cardiomyocytes maintain a 10 000-fold electrochemical gradient of calcium across their plasma membrane, which is one of the highest in the body, augmentation of cardiomyocytes by calcium is a plausible explanation for high sensitivity of cardiomyocytes to PFA damage. In this context, Maizels and colleagues successfully demonstrated in human-induced pluripotent stem cells how PFA damage to cardiomyocytes can be enhanced through electroporation Ca^2+^-sensitization.^38^

Many other biological mechanisms could potentially explain differences observed between cardiac tissue and other tissue types. For example, it has been shown that PFA can alter the metabolic activity of cardiomyocytes by affecting electron transport in the mitochondria^41^ and that the effect is sensitive to PFA voltage and energy dose.^42^ Comparative studies between different tissue types are needed to clarify the role of such effects in preferential cardiac ablation.

Additionally, most of the current knowledge comes from basic *in vitro* studies. However, some of the biological components that may explain PFA cell death mechanisms such as effects on microcirculation and vasculature, regenerative capabilities, inflammatory and immune responses, etc., are tightly linked to *in vivo* conditions and cannot be replicated *in vitro* increasing the complexity of mechanistic studies.

#### Tissue regeneration

Besides biophysics and cell biology, the third reason for the observed preferential effects of PFA on cardiac tissue is that cardiac tissue does not regenerate after electroporation injury as many other tissues do. While radiofrequency ablation (RFA) destroys not just cells but also the extracellular matrix, independent of tissue type, which induces non-specific tissue damage, IRE preserves the extracellular matrix and other tissue components such as the microcirculatory system.^6,8^ In this way, IRE decellularizes the target tissue while keeping the patency of extracellular structures.^43^ The most important consequence of this phenomenon is that it can be applied to nearby critical structures such as vessels, nerves, and others as demonstrated in cancer applications.^8^

Decellularized scaffolds left after IRE can potentially be repopulated with cells, and they are widely studied for applications in regenerative medicine.^44,45^ Previous studies have shown how the preservation of the extracellular matrix after IRE enables cell regeneration and tissue healing in the liver,^46^ blood vessels,^27,47,48^ nerves,^49^ oesophagus,^50–53^ etc. Depending on the electric field application conditions and tissue under study, some works reported full regeneration of the full tissue or partial regeneration of some tissue layers.^47^

Adult mammalian cardiomyocytes are widely regarded as terminally differentiated with minimal regenerative capacity, and cell loss is typically not replaced by new contractile myocardium. Reviews of myocardial biology note that adult cardiomyocytes exhibit extremely low rates of cell turnover and respond to injury primarily by scar formation rather than regeneration.^8^ It is not surprising, then, that cardiac tissue has not been found to spontaneously regenerate after IRE.

For other types of tissues, the ability to regenerate after PFA has been convincingly demonstrated. Nerves can achieve complete functional recovery within weeks.^49^ Treated liver tissue was replaced by normal hepatocytes with minimal scarring within 3 weeks.^54^ For skeletal muscle, PFA can create massive myofibre necrosis that is followed by efficient muscle regeneration.^55^ Collectively, these examples demonstrate that the very limited regeneration of myocardium is part of the reason for PFA acting preferentially on cardiac tissue (see *Figure 1*).

Relevant to cardiac applications, several preclinical works have shown how oesophageal tissue can heal and regenerate after PFA.^50,51,53^ Although the process of tissue regeneration after IRE is still poorly characterized, some preclinical studies have shown how the tissue microenvironment, including nerves, lymph vessels, and blood vessels, is preserved providing a necessary functional framework for cell regeneration. Production and migration of progenitor cells have been reported in histological examinations^52^ likely facilitated by differential cell death pathways activated like pyroptosis or necroptosis. Finally, although some studies have reported reduced inflammation in cardiac tissue after PFA compared to RFA,^56,57^ it is well known that IRE can produce a local innate immune system activation. The specific characteristics of this non-thermal immune response, including the release of damage-associated molecular patterns could also play an important role in facilitating tissue regeneration.

### PFA in different cardiac substrates

Despite its potential, several gaps in knowledge regarding PFA in different substrates remain. Evidence about the chronic durability of PFA lesions has been extensively developed for pulmonary veins (PVs) but is still scarce in thick, extra-PV substrates. Remapping studies have shown very high rates of reconnection when using PFA tools designed for PVI in targets like the mitral isthmus (5.5% durability), left atrial appendage (4.6%), and the coronary sinus (1.3%).^57^

#### Heterogeneity of myocardial tissue

As previously explained, local electroporation depends on the redistribution of the electric field at a tissue level, which is intrinsically linked to the complex, non-uniform nature of the cardiac substrate. Cardiac myocardium is not a simple syncytium but a 3-dimensional entanglement of compact muscular bundles, adipose tissue, vascular networks and collagen-rich areas. This heterogeneity varies from patient to patient and from region to region and is present both in ventricles and atria alike. In the posterior mitral isthmus region, for example, compact myocardium consistently ranges from 2 to 4 mm in thickness, but it is frequently interspersed with non-conductive epicardial fat that can reach depths approaching 1 cm. In diseased myocardium, structural complexity is further increased by the presence of fibrosis but also potential fat or calcifications.

This structural heterogeneity creates significant challenges for energy delivery. While traditional thermal modalities are impeded by the thermal and electrical insulating properties of fat and fibrotic tissue and the convective cooling of vessels, PFA effects depend mostly on the passive electrical properties of these different components. Electric field distribution for non-homogenous tissues will depend on the electrical conductivity transitions between adjacent tissues. Transitions in conductivities between myocardium, fat or connective or fibrotic tissue will create local modifications of the electric field intensity amplifying or reducing the electric field locally (see *Figure 2*).

**Figure 2:**
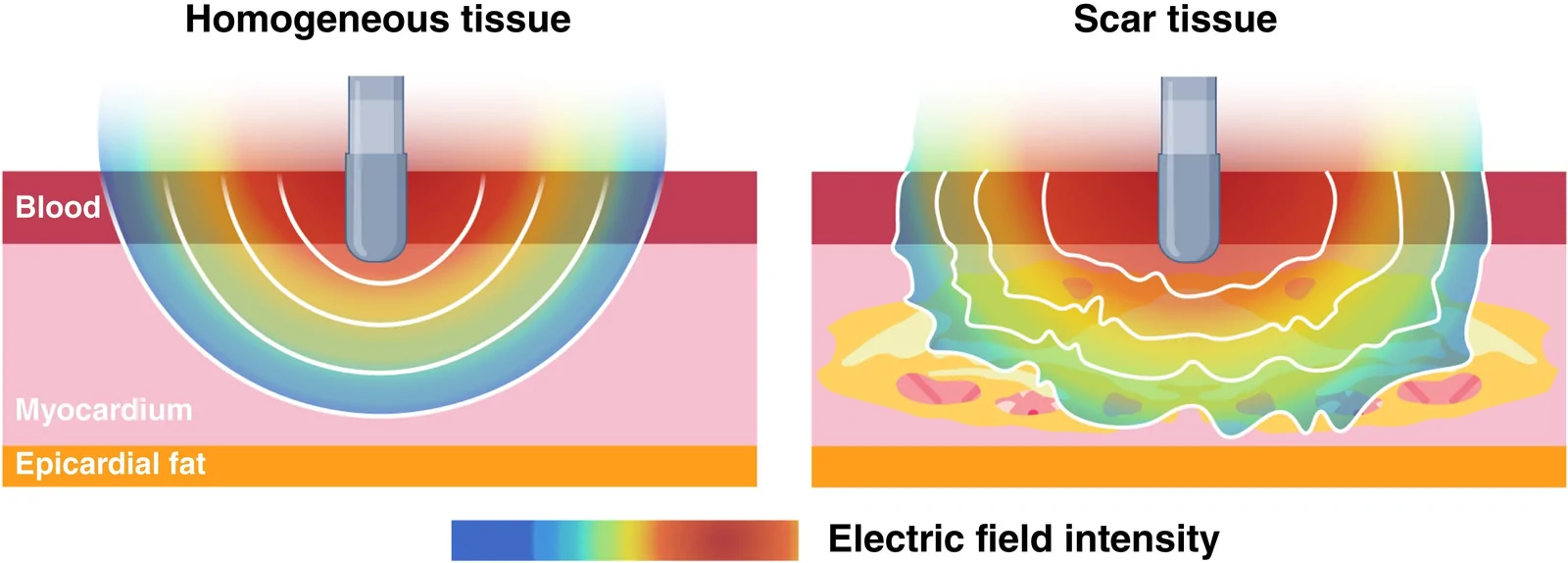
Schematic comparison of electric field distribution in homogenous vs. scar cardiac tissue. Computational models predict electric field penetration even with a dense scar tissue. Presence of fibrosis and fat can produce localized distortions of electric field distribution. Partially adapted from ref.^58^.

#### Ablation in healthy vs. diseased myocardial tissue

The effects of PFA across varying myocardial substrates have been examined in computational simulations, preclinical models (mostly swine),^59–61^ and clinical studies. Computational modelling suggests that while fibrotic tissue has a negligible impact on electric field distribution (and hence on lesion formation), the presence of intramyocardial fat within the scar significantly alters lesion morphology (see *Figure 2*).^58^

Preclinical studies in porcine models of chronic myocardial infarction confirm that PFA successfully penetrates both ischaemic and iatrogenic RFA scars. In these models, lesion depth remained consistent between infarcted border zones (5.9 ± 1.0 mm) and healthy remote myocardium (5.7 ± 1.3 mm; *P* = 0.53). Furthermore, the lethal electric field threshold required for irreversible injury appears similar in both healthy and infarcted porcine hearts.^62^ ‘Redo’ models further indicate that PFA is highly effective when ablating over chronic RFA scars, producing lesions that are significantly deeper and more uniform than repeat RFA applications (7.1 ± 1.3 mm vs. 3.9 ± 1.6 mm; *P* < 0.01).^63^

Early clinical experience shows the important potential of focal PFA for scar-related ventricular tachycardia ablation. Case reports and clinical series show high success rates in achieving transmural tissue homogenization that is often unattainable with conventional thermal energy.^64–66^ These results have been achieved in a variety of settings, underlying substrates and as bailout therapy after failed RFA, confirming the value of PFA in this setting.

##### Ablation around fat and vascular structures

As explained before, the biophysical interactions between PFA and different tissues are governed by differences in electrical conductivity. Adipose tissue has a conductivity 10- to 20-fold lower than healthy myocardium producing a local alteration of the electric field.^67^ Although it could seem counterintuitive, computational modelling shows that fat serves as an ‘attractor’ for the electric field, around which field strength tends to peak. This creates localized ‘hot points’ of high electric field strength in the lateral areas of fat deposits, while ‘cold points’ appear at the entry and exit points of the electrical current.^58^ It is important to highlight that the effects of PFA depend on the electric field intensity, which is directly proportional to the current density and inversely proportional to the conductivity. This explains how electric field can be higher in areas of low conductivity.

Other structures in the tissue such as blood vessels can also significantly modify the electric field distribution. Due to the higher conductivity of the blood inside the vessels, there will be a local modification of the electric field around the vessel with areas of low and high electric field, which may contribute to inhomogeneous ablation of target myocytes residing in the immediate vicinity of these sinks. This was modelled and experimentally confirmed in liver tissue.^68,69^ On the contrary, the vascular wall has relatively lower conductivity, which may create electric field peaks in the transition areas between tissue and blood.^68^

#### Comparison with RFA

Radiofrequency ablation lesions are often limited by the insulating effects of intramyocardial collagen and fat. In preclinical comparisons within heterogeneous scar, PFA produced lesions that were deeper and more transmural than those of RFA (6.4 vs. 5.4 mm; *P* = 0.024), effectively eliminating viable myocytes separated from the catheter by adipose and fibrotic barriers.^61^ Furthermore, PFA is not subject to the ‘heat-sink’ effect of local blood flow or poor thermal conductive properties of scar tissue, which significantly blunts the intramyocardial temperature rise during RFA in infarcted tissue. Recent preclinical studies have explored the potential of colocalized sequential combinations of RFA and PFA as a strategy to increase lesion depth in healthy ventricular myocardium with mixed results.^70,71^ Although potential mechanisms are still not demonstrated and available evidence is still scarce, future research should explore if scar tissue could be an interesting target for colocalized energy combinations.

### Lesion formation mechanisms

Pulsed field ablation has introduced a novel paradigm for myocyte injury during catheter ablation. Unlike conventional thermal ablation modalities, PFA employs high-intensity electric fields and is characterized by distinct mechanisms of cell death, unique histopathological features, and a different temporal course of lesion maturation. A comprehensive understanding of the *in vivo* processes underlying lesion formation after PFA, as compared with conventional thermal ablation, is essential to optimize both the safety and efficacy of this technology in clinical practice. In this section, we review the acute, subacute, and chronic phases of the lesion formation following PFA and compare these findings with those observed after RFA.

#### Acute observations

The mechanisms of cell death induced by PFA are thought to include necrosis and apoptosis, among several other potential pathways.^34^ However, the temporal evolution of these processes and their relative contribution to contemporary PFA waveforms used in cardiac ablation remain incompletely characterized. Compared with RFA, PFA is believed to preferentially induce non-thermal apoptosis-like delayed cell death, which renders PFA lesions difficult to identify immediately after energy delivery as no changes are observed macroscopically. Over the subsequent 2–4 h, PFA lesions gradually become discernible if a metabolic activity staining as triphenyltetrazolium chloride is used (*Figures 3A* and *4A*). This temporal evolution is also relevant for lesion assessment using intracardiac echocardiography (ICE). Unlike RFA, maximum echogenic changes cannot be visualized on ICE immediately after application and become progressively more apparent few minutes after PFA with a peak at up to 60 min (*Figure 3B*).^70^ It has been suggested that at least 24 h are required for acute PFA lesions to fully mature, and an animal study has demonstrated that apoptosis becomes apparent by 6 h and peaks at 24 h post-ablation.^71^ Consistent with these findings, clinical studies have reported that myocardial injury biomarkers reach their maximum levels within 3 h while NT-proBNP levels peaked at 24 h following PFA procedures.^77,78^

**Figure 3:**
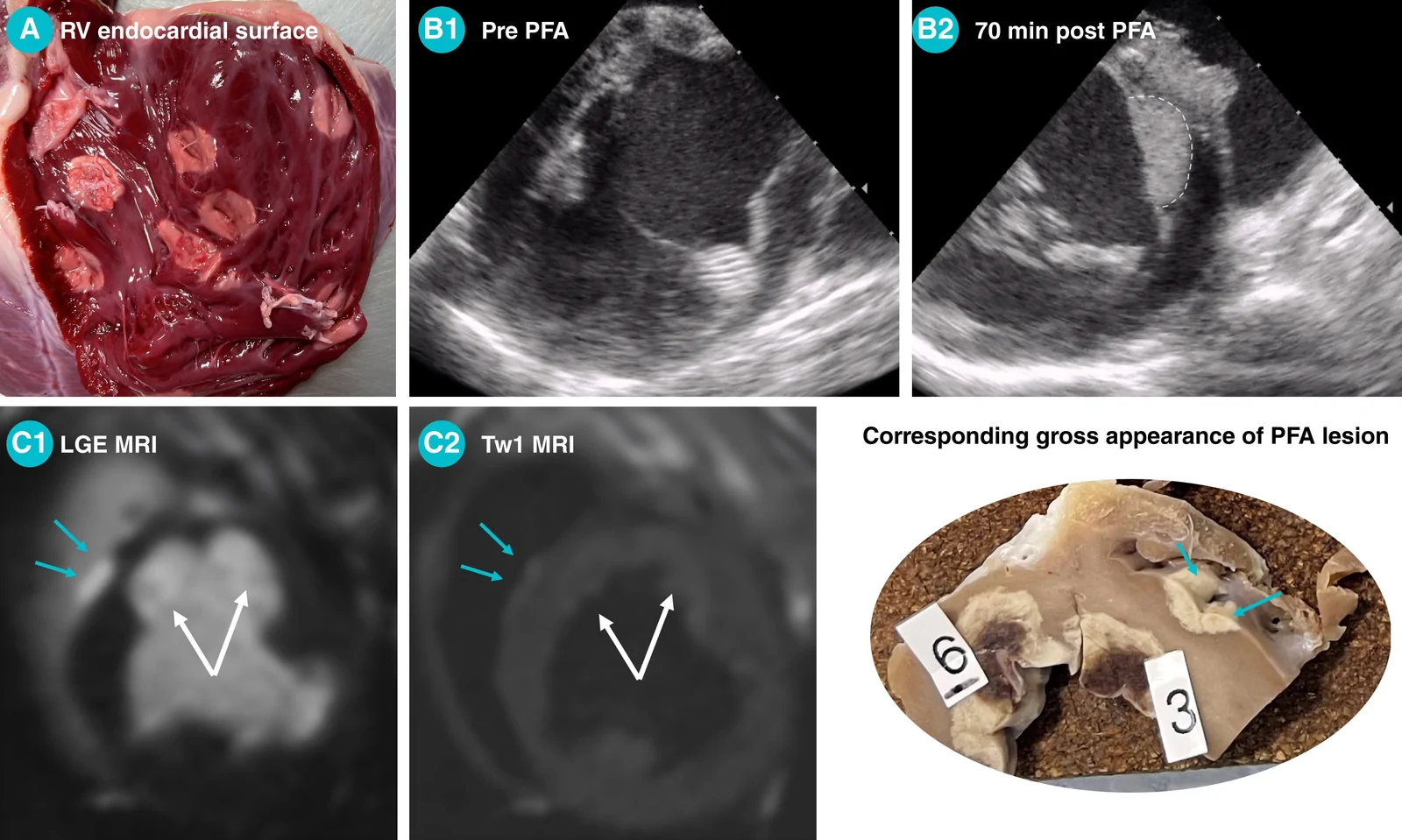
PFA lesions; histology, intracardiac echocardiography, and MRI. (*A*) Gross findings of PFA lesions on the right ventricular surface 24 h after PFA, stained with triphenyltetrazolium chloride. Uniform lesion formation is observed even in tissue with relatively developed trabeculae, with clearly demarcated borders. In addition, intralesional haemorrhage is minimal. Modified from ref.^59^. (*B*) ICE images obtained before PFA lesion formation and 70 min after PFA show a hyperechoic lesion becoming visible at 70 min. PFA lesions are difficult to visualize immediately after ablation on ICE. Modified from ref.^59^. (*C*) MRI images acquired 24–48 h after PFA demonstrate no obvious findings of microvascular obstruction in PFA lesions, with homogeneous late gadolinium enhancement and minimal enhancement on T1-weighted images. Black arrows indicate right ventricular lesions, and white arrows indicate left ventricular PFA lesions.

Histologic reports describing tissue changes within the first 1–2 h after PFA are limited. An important aspect to consider is that observed histological changes may differ between areas very close to the application catheter, where the electric field is maximum, and areas at a certain distance from it.^79^ Available studies have noted that the most distinctive observation of PFA lesions is the almost immediate development of contraction bands and interstitial oedema accompanied by nuclear condensation, cardiomyocyte swelling, and inflammatory cell infiltration with some areas of myocardial necrosis and haemorrhage.^80,81^ Electron microscopy studies have likewise demonstrated some distinct features of PFA lesions compared to RFA with basement membrane disruption, interstitial oedema, and bleeding notably reduced in comparison to the corresponding RFA. Other features such as nuclei deformation with condensed chromatin and changes in mitochondria (swollen with focal deposits and in clustered arrangements) were still apparent.^82,83^ Compared with RFA, PFA lesions are associated with milder oedema, reduced intravascular erythrocyte accumulation, and less erythrocyte extravasation (*Table 1*). Notably, even at the ultrastructural level, PFA lesions exhibit an abrupt transition in the degree of myocyte damage, reflecting the sharp demarcation characteristic of PFA-induced injury.^82^ The state of increased cell membrane permeability after IRE can span from minutes to hours until cell death is observed.^84^ Indeed, myocyte membrane structure and organelle structure progressively deteriorate by at least 4 h post-ablation in the electron microscopic study.^82^

**Table 1:** Comparative summary of main histological findings between PFA and RFA at 1 and 4 h after ablation

	1 h PFA	1 h RFA	4 h PFA	4 h RFA
**Cardiomyocyte disruption**	++	+	+++	++
**Mitochondria misalignment, swelling and focal deposits**	+	+	++	++
**Membrane disruption**	+	+	+	+++
**Oedema**	+	++	++	++++
**Intravascular erythrocyte accumulation**	−	+	−	+++
**Extravasation of erythrocyte**	−	+	−	+++

(+) and (−) symbols indicate presence or absence of specific findings.

PFA, pulsed field ablation; RFA, radiofrequency ablation.

#### Subacute observations

Radiofrequency ablation primarily induces coagulative necrosis as the dominant mechanism of cell death, which is followed by a marked inflammatory response characterized by macrophage infiltration and subsequent fibroblast proliferation. In contrast, PFA can also promote delayed cell death pathways such as apoptosis. Histological findings at 24–48 h after PFA application demonstrate reduced haemorrhage as compared to RFA, suggesting less extensive vascular damage^85^ (*Figures 3A* and *4B*). Lesion borders were well-demarcated, with immunohistochemical analysis at 48 h showing an abrupt loss of troponin I expression at the lesion margins, along with blurring of cell membrane staining.^59^

**Figure 4:**
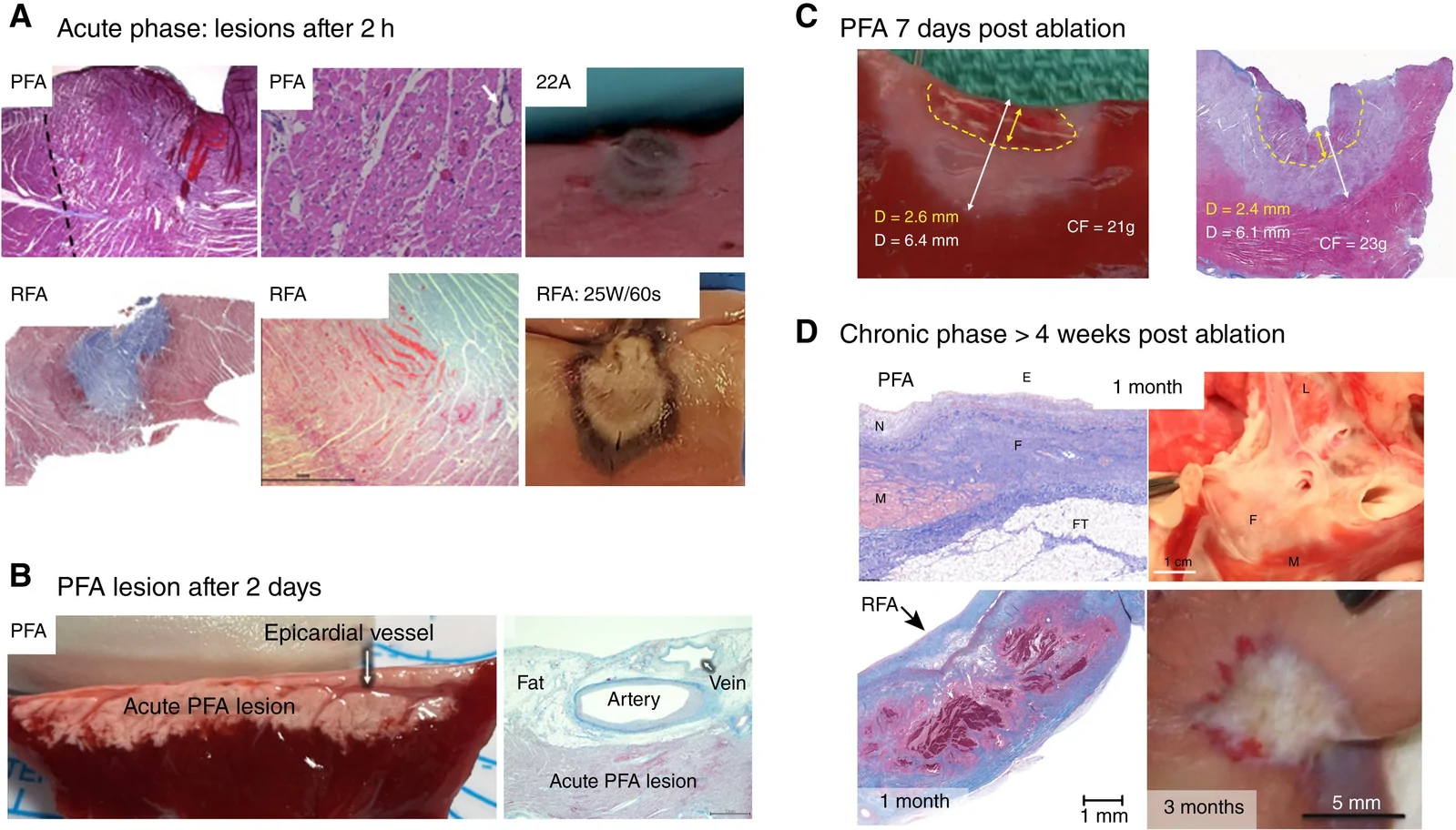
Different stages of lesion formation after PFA and RFA. (*A*) Acute phase: Histologic (left and middle) and macroscopic images (right) of PFA (monopolar, biphasic, top row) and RFA (bottom row) delivered via a 3.5 mm focal ablation catheter. Note the subtle macroscopic features of the acute PFA lesion as compared with the clearly demarcated coagulative RFA lesion. Histopathology shows well-demarcated non-coagulative acute myocardial damage in the ablated area (right of dashed line) with intact vessels (arrow). In contrast, histopathology of RFA lesions shows coagulation necrosis (blue) surrounded by haemorrhagic contraction band necrosis (dark purple). Modified from ref.^75^. (*B*) PFA lesion 2 days after epicardial delivery using a 9 mm lattice-tip catheter showing a homogeneous, sharply demarcated lesion with preservation of intralesional fat and blood vessels. Modified from ref.^59^. (*C*) Gross pathology (left) and histopathology (Masson’s trichrome staining) of a PFA lesion created with an investigational 2.3 mm tip focal ablation catheter using an output of 2.0 kV. The lesion is composed of two areas. The central, shallower area (yellow dotted line) shows evidence of coagulative necrosis, indicating a minor thermal effect in the lesion’s centre using this investigational catheter and ablation settings. The remaining, larger area of the lesion (white arrow) exhibits homogeneous fibrosis consistent with pulsed field ablation injury. ×20 magnification. Modified from ref.^89^. (*D*) Chronic phase >4 weeks post-ablation. The top row shows a PFA lesion at the opened RSPV macroscopically (right) and in histopathology (left) at 4 weeks post-ablation using a nine-electrode circular ablation catheter (bipolar PFA, 1.5 kV). Both images illustrate homogeneous replacement fibrosis. Histopathology confirms transmural fibrosis without residual necrosis or inflammation. In comparison, RFA lesions show islands of persistent cardiomyocyte necrosis in the lesion centre 4 weeks post-ablation (left). The black arrow indicates endocardial hyperplasia. The right image shows a fully mature RFA lesion 3 months post-ablation at gross necropsy. The lesion appears more inhomogeneous with a pale/brown lesion core with dense fibrosis, surrounded by a purple partially haemorrhagic rim. E, endothelium; F, fibrotic lesion; FT, fat tissue; L, lung; M, myocardium (untreated); N, neointima; RPSV, right superior pulmonary vein. Modified from refs ^76,161,162^.

Inflammatory response also evolves with time, with inflammatory cells such as lymphocytes, macrophages, and multinucleated giant cells observed in 7-day samples and reduced over time.^86^ Seven days after PFA using an investigational 2.3 mm focal ablation catheter with moderate contact force, lesions displayed a large area of homogeneous collagenous replacement fibrosis with a shallow central zone identified as potential thermal injury (*Figure 4C*).^73^ In an animal study comparing tissue inflammation between RFA and PFA at 2–4 weeks after ablation, inflammation within PFA lesions has been demonstrated to be substantially milder as compared to RFA. Parameters including necrosis, inflammation, oedema, haemorrhage, and thrombus formation were more frequent and severe in RFA lesions.^87^ In contrast, PFA lesions evolve into homogeneous fibrotic tissue without residual viable myocardium, while non-myocardial structures such as blood vessels and nerves are preserved (*Figure 4B*).^87^ Mineralization is also a common observation at subchronic timepoints, suggesting an inflammatory response to calcium-rich cell debris.^85,86^

On cardiac magnetic resonance imaging, PFA also displays distinct features suggesting different tissue damage and healing processes. RFA lesions typically exhibit high signal intensity on T1-weighted images due to iron oxidation induced by thermal injury (oxidation of ferrous (Fe^2+^) to ferric (Fe^3+^) iron within haemoglobin and myoglobin occurs at temperatures of 55–65°C, whereas this finding is less pronounced in PFA lesions (*Figure 3C*). With gadolinium-enhanced imaging, RFA lesions often demonstrate microvascular obstruction because vessels are occupied by erythrocytes, whereas PFA lesions more frequently show homogeneous late gadolinium enhancement (*Figure 3C*).^62,88^

#### Chronic observations

Histologically, mature lesions represent sharply demarcated, homogenous areas of replacement fibrosis with preserved tissue architecture and spared non-myocardial structures such as nerves and blood vessels. Preclinical studies have demonstrated that PFA lesions are mostly mature by 3 weeks post-ablation, only showing a further increase in collagen density at later time points.^80^ In contrast, RFA produces more extensive central tissue damage, where coagulation necrosis and non-selective protein denaturation disrupt tissue architecture and vasculature. Consequently, RFA lesions often mature into less homogeneous scars with chronic inflammation and persistent necrosis present even at 1 month post-ablation (*Figure 4D*).^89^ Compared with thermal ablation, a limited inflammatory response is observed that typically resolves within 30 days after PFA in preclinical models.^86^

Tissue contraction during scar formation after RFA is a well-recognized cause of PV stenosis when ablation is performed within the PVs. In contrast, due to its non-thermal mechanism, preservation of extracellular matrix and distinct inflammatory response, PFA has not been associated with PV stenosis, even when applied deep within the PVs in preclinical studies.^90^ PFA has been reported to induce less PV fibroblast proliferation compared with thermal energy-based ablation.^91,92^ These favourable safety findings have been confirmed in large clinical registries.^93^ In light of these data, direct ablation of arrhythmogenic myocardial sleeves within the PVs has recently been reintroduced in novel PFA protocols to improve PVI durability.^94^ However, the safety of PFA for PV stenosis in patients with a prior history of thermal ablation, particularly in those who have already developed PV stenosis as a consequence of previous thermal energy delivery, requires further investigation.^95^ Furthermore, the reversible ‘stunning effect’ after PFA raised questions about potential lesion regression during maturation. However, clinical data from invasive remapping studies demonstrate that lesion size, as assessed by low-voltage areas in 3D electroanatomical mapping, does not significantly decrease until 3 months after PFA using a pentaspline catheter.^96^

Post-ablation inflammation and remodelling processes are integral to injury healing and lesion maturation but may trigger early arrhythmia recurrences that do not necessarily predict long-term outcomes. Traditionally, arrhythmia recurrences during the so-called ‘blanking period’ have not prompted long-term treatment decisions such as repeat ablation. This blanking period was historically defined as the first 3 months following ablation, although recent guidelines recommend shortening it to 8 weeks.^97^ This new definition has also been challenged, as arrhythmia recurrences occurring more than 1 month after thermal ablation strongly predict poor long-term success.^98^ The advent of PFA, with its reduced inflammatory response and rapid lesion formation, provides further rationale for a more nuanced definition of the blanking period, as the timing and significance of early recurrences may differ following PFA.^98^

## Part II: PFA effects on nerves

### Effects of PFA on nerves

During PFA treatments, nerve structures surrounding the target ablation area can potentially be exposed to relatively high electric field intensities due to their relative anatomical position with respect to cardiac tissue.

#### Biophysics of PFA effects on nerves

The two most important effects that PFA can exert on nerves are either electrical stimulation or electroporation (reversible or irreversible). The mechanism in both cases is that an externally applied electric field induces a transmembrane potential distribution along the axons; wherever the local transmembrane potential exceeds the stimulation threshold for a sufficient time, the axon is stimulated; wherever it exceeds the electroporation threshold, electroporation occurs (see *Figure 5*).^99,100^ The most important difference between these two phenomena is the minimum electric field necessary to induce one or other effect (the electric field threshold) being the threshold for stimulation considerably lower than the threshold required for electroporation.

**Figure 5:**
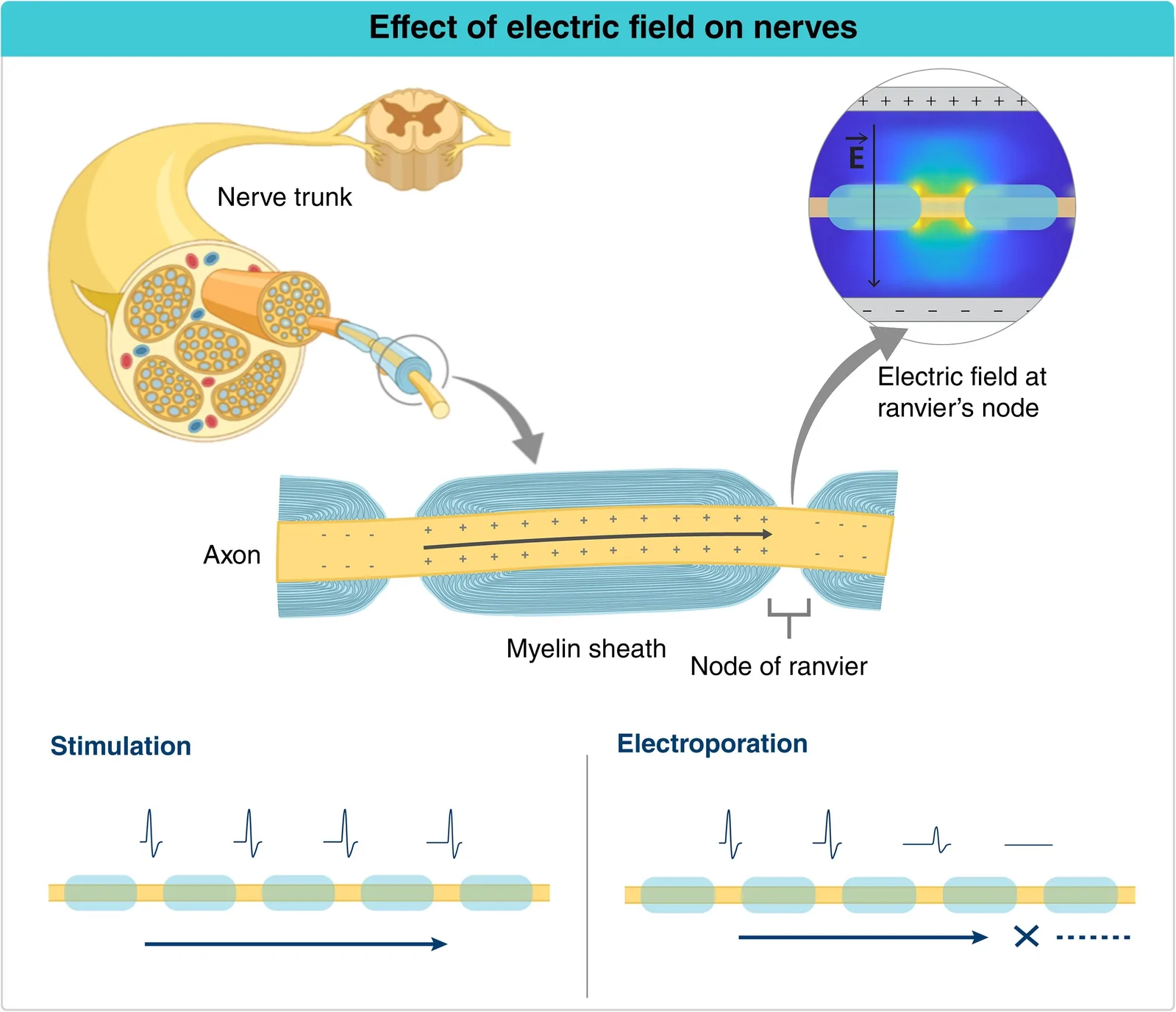
Schematic representation of the interaction of an electric field with a myelinated axon. Electric field concentrates in the unmyelinated areas leading to depolarization and stimulus propagation or local cell membrane electroporation leading to a possible transient conduction block.

Nerves contain multiple fibre bundles, which are in turn composed of individual nerve fibres. Each nerve fibre contains an axon, i.e. a long, slender cellular extension of a neuron optimized for electric signal conduction. For faster signal transduction, many axons are enveloped in myelin sheaths, except at isolated nodes of Ranvier, and this anatomy affects their response to the high-amplitude fields that are applied during PFA. Like cardiomyocytes, or other excitable cells, axons are excited when their membrane is depolarized to the point at which the voltage-gated channels open. Since multiple axons run in parallel to the same destination, there can be a graded effect depending on the distance from the high electric field source ranging from electrical stimulation producing recruitment of axons to electroporation potentially producing partial nerve block. Another important factor to consider for long nerve fibres is electric field direction with respect to the fibre. The transmembrane voltage induced by an electric field in the direction of the fibre is much higher than the transmembrane voltage induced by a transverse electric field. However, the membrane charging time is also significantly longer for longitudinal orientations.^101^ For long-duration electric field pulses, longitudinal orientation is more effective to excite long fibres but when pulse duration is below a certain value (< 100 ns) the transverse orientation can be more effective at generating action potentials with a lower electric field intensity.

##### Stimulation

When considering off-target neuromuscular side effects of PFA, inadvertent stimulation of myelinated nerve fibres is of particular concern because myelinated axons exhibit lower extracellular stimulation thresholds and are preferentially excited by high spatial gradients of applied electric fields.^102,103^ Activation of these fibres mediates acute, readily apparent effects, such as sharp pain and skeletal muscle contraction, through recruitment of fast sensory and motor axons.^104,105^ In contrast, unmyelinated autonomic fibres generally exhibit higher activation thresholds and slower conduction velocities, and their activation typically produces delayed or less immediately perceptible physiological effects.^102,106^

Myelin has a high electrical resistance and low capacitance, which markedly attenuate transmembrane polarization along internodal regions; as a result, myelinated axons are largely insulated from externally applied electric fields except at the nodes of Ranvier, where myelin is absent.^102,107^ At the nodes of Ranvier, geometric concentration of transverse current leads to enhanced induced transmembrane voltage, while voltage-gated sodium channels are clustered at densities orders of magnitude higher than in internodal membranes.^104,108^ Consequently, extracellular electrical stimulation of myelinated axons is generally initiated at nodes of Ranvier.^103,104^

Experimental studies have demonstrated that peripheral nerves can be stimulated using a wide range of pulse durations and waveforms. Classical nerve stimulation has most commonly employed millisecond- to hundreds-of-microsecond-duration pulses.^104^ However, repeated stimulation can also be achieved with short microsecond pulses^102^ and even pulses as short as 12 ns, without any apparent damage.^109^

##### Electroporation

Like any other lipidic bilayer, the cell membrane of nerves can also undergo electroporation if the local electric field is high enough to exceed the electroporation threshold. Theoretically, different effects could be expected in different areas of nerves based on local membrane curvature, size, and electrical field distribution. In this sense, the soma (cell body) of neurons should display a higher vulnerability than the axons. However, there is no solid experimental evidence demonstrating this. In myelinated axons, the myelin sheath markedly attenuates transmembrane polarization along internodal regions, externally applied electric fields also preferentially polarize nodal membranes, rendering nodes of Ranvier the most susceptible sites for electrically induced membrane perturbation, including electroporation, in myelinated axons.^102,103,110^ This nodal vulnerability reflects both the absence of myelin and the concentration of electric stress at discrete, highly excitable membrane regions (see *Figure 5*).^103^ Consequently, electroporation of axons is expected to be a localized phenomenon occurring preferentially at the nodes of Ranvier more than a generalized effect across the entire membrane of the axon.

The state of increased permeabilization of the cell membrane due to electroporation can produce nerve conduction abnormalities, transient nerve dysfunction has been reported including nerve conduction velocity reduction without complete nerve block, with spontaneous recovery observed in clinical case reports.^111–113^ Reversible electroporation typically resolves within minutes to hours as membrane integrity is restored,^23^ and more extensive yet non-degenerative membrane disruption involving multiple nodal or paranodal regions may recover over days to weeks.^49^

Permanent structural nerve injury is generally associated with more extensive or sustained membrane damage, which may extend beyond isolated nodal membranes to involve multiple adjacent nodes and/or disrupt paranodal axo-glial junctions and myelin integrity.^114^ Such disruption can compromise ionic homeostasis and axonal transport, ultimately leading to axonal degeneration, including Wallerian degeneration in severe cases with subsequent regeneration.^49,115,116^ Experimental observations have described preservation of endoneurium and epineural extracellular matrix architecture and proliferation of Schwann cells suggesting the potential for axonal regeneration at later stages as observed in different studies (see *Figure 6*).^49,115^ These observations are radically different to what is observed in thermal ablation modalities, where nerve extracellular nerve structures can also be damaged.

**Figure 6:**
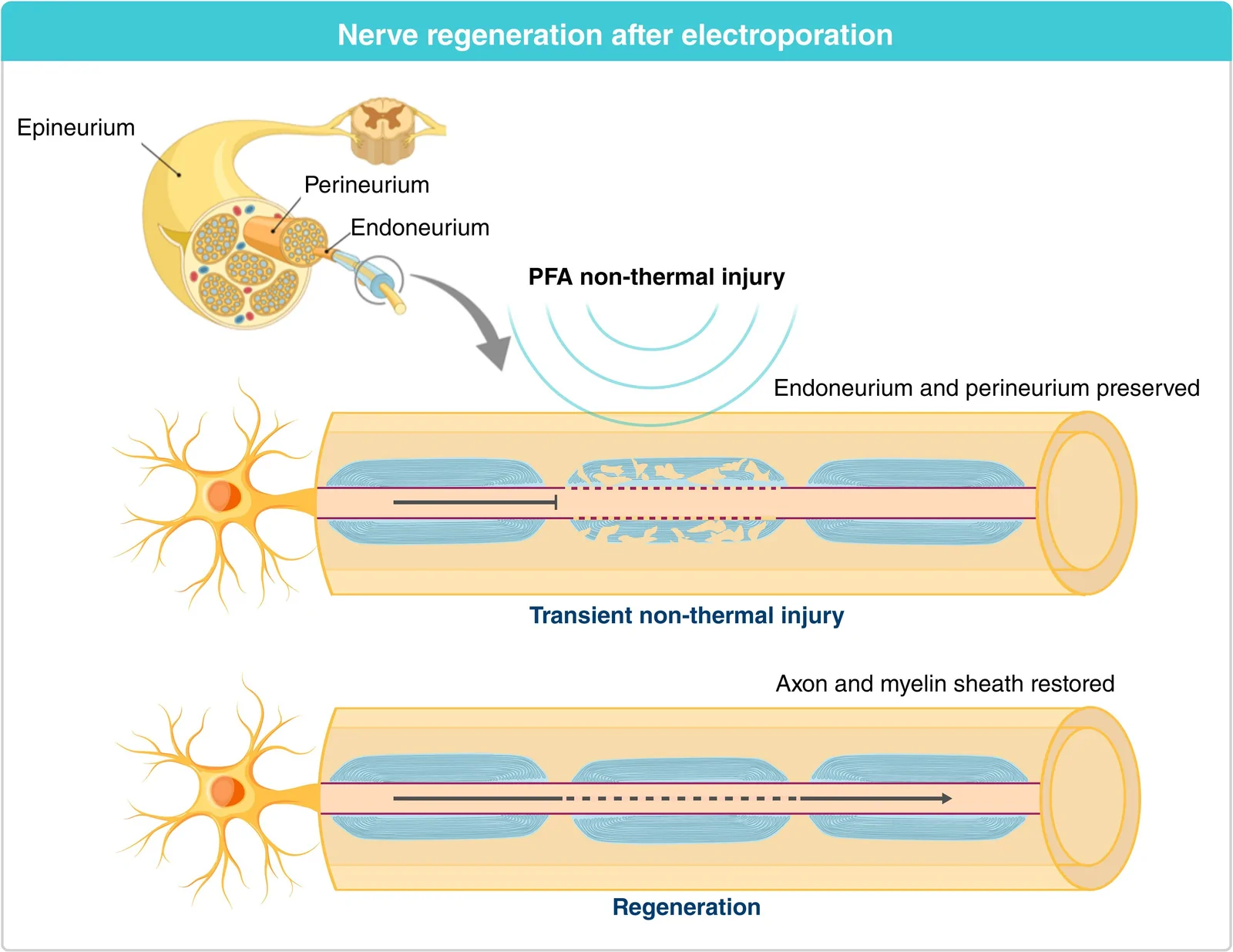
Possible nerve regeneration mechanisms after electroporation. Axons and myelin sheath can be transiently disrupted after exposure to very strong electric fields. However, nerve structures such as the endoneurium and perineurium are not thermally damage in PFA enabling subsequent nerve regeneration mechanisms and nerve function restoration.

#### Effects of PFA on the phrenic nerve

Among the different nerve structures that may be affected by PFA, the phrenic nerve is particularly relevant, especially in AF ablation because of its anatomical proximity to the right PVs and the superior vena cava. In the context of thermal ablation technologies, the term phrenic nerve injury (PNI) has traditionally been used to describe any alteration in phrenic nerve function, whether transient or permanent. With the emergence of non-thermal PFA, it may be appropriate for the scientific community to reconsider the continued use of the term injury. This terminology implies structural damage to the nerve, which may not accurately reflect the mechanisms associated with PFA. Consequently, alternative terminology—such as phrenic nerve dysfunction—may better characterize functional alterations observed with this technology.

##### Transient effects

The most common effect observed on the phrenic nerve during PFA procedures is electrical stimulation. Electric field intensities above the simulation threshold can reach the phrenic nerve particularly in the right PVs where the distance between the catheter and the phrenic nerve is lower. Local nerve stimulation can lead to abrupt diaphragmatic contractions during each PFA application without further clinical implications. However, studies have also reported a very low incidence of other transient phrenic nerve conduction disturbances.

Observations with traditional thermal ablation have reported transient effects on the phrenic nerve, displaying temporary conduction block with either immediate intraprocedural or delayed recovery within days to weeks. The transient nature of these effects for the specific case of cryoablation has been mainly attributed to local axonal conduction block without structural axonal disruption due to direct cooling of the phrenic nerve due to thermal spread.^117^ Other suggested mechanisms include transient reduced blood supply to the nerve due to microvascular constriction or injury.^118^ Also, local inflammation and tissue oedema may compress or irritate the phrenic nerve producing delayed effects.^119^

In the case of PFA, transient effects on the phrenic nerve leading to temporary conduction block have also been reported. Due to the completely different nature of PFA energy, the behaviour and mechanisms behind the observed transient effects are also different. First, according to clinical observations, the dynamics of recovery seem to be much faster than for thermal injury. Detailed compound motor action potential characterization revealed a rapid recovery phase 1–2 min after PFA applications with complete (53%) or partial (47%) signal recovery without X-ray changes in either case.^120^ Preclinical data also showed similar recovery dynamics in swine in a dose–response manner.^121^

The most plausible mechanism explaining the rapid transient effects observed after PFA is reversible electroporation. Electroporation will create a state of high membrane permeability of axons producing a transient electrical conduction block. Reversible electroporation generally resolves within seconds to minutes, although in some experimental observations it may persist for several hours,^23,122,123^ which appears to be consistent with the observed time course of phrenic nerve functional recovery. Once cell membrane reseals, local conduction capability is restored without any other relevant structural changes to the nerve. Although not experimentally proven, other alternative mechanisms such as oedema or transient inflammation cannot be ruled out as contributing factors to some more durable, still transient, observations which may not be possible to explain by reversible electroporation.

##### Chronic effects

Traditional thermal ablation technologies have demonstrated that permanent PNI is an important complication to consider when evaluating new ablation technologies with persistent damage, leading to hemidiaphragmatic paralysis, occurring in about up to 0.3–1.5% of cases depending on the technology used.^124,125^

In the context of PFA, the incidence of permanent phrenic nerve paralysis appears to be extremely low based on current clinical trials and large registries with only isolated case reports described. Preclinical work already suggested this low vulnerability of the phrenic nerve to PFA acutely with no chronic damage observed.^121,126,127^ However, more recent preclinical literature with the ballon-in-basket PFA catheter reported some instances of minimal axonal degeneration with multifocal myelin sheath and axonal swelling at 34 ± 4 days even without noticeable functional impact.^128^

Clinical data have also shown the remarkable safety profile of PFA. In the MANIFEST-PF registry including 1568 patients ablated with the pentaspline catheter, only one case of persistent PNI was reported (0.06%).^129^ Similarly, the large MANIFEST-17 K study involving more than 17 000 patients reported no cases of permanent PNI and only 0.06% transient phrenic nerve dysfunction.^130^ A recent case report described persistent right phrenic nerve palsy after PVI, presenting with dyspnoea and elevation of the right hemidiaphragm detected shortly after the procedure and persisting 8 weeks post-procedure without subsequent follow-up information.^131^ Another report highlighted a similar complication with a circular PFA catheter attributed to an ablation delivered in close proximity to the pericardiac phrenic bundle.^132^ These cases suggest that although the phrenic nerve may be significantly less susceptible to PFA effects, permanent phrenic nerve dysfunction may occur even at extremely low rates.

##### Practical clinical considerations

The potential effects of pulsed electric fields on the phrenic nerve largely depend on the cumulative electric field dose to which the nerve is exposed. Basically, this will be defined by the electric field strength, the total number of applications that may overlap in a certain region, and importantly, by the intrinsic waveform characteristics of each particular PFA system. Thus, comparing the safety profile of different PFA systems on the phrenic nerve based on simple variables as the output voltage or distance is not possible and robust clinical data should be the standard to compare.

From a clinical practice perspective, it seems extremely challenging to generalize best practices for phrenic nerve safety. Some current PFA systems do not include any specific phrenic nerve safety recommendations, while others recommend reducing output voltage or catheter repositioning to mitigate potential risks. Overall, it seems reasonable to think that as the observed effects follow dose–response behavior, being mindful of catheter position and being vigilant of acute responses could be adopted as general phrenic nerve safety best practices.

#### Specific effects of PFA on the cardiac ganglionated plexi

##### Introduction

In this section, the effects of PFA on the autonomic nervous system will be analysed. Across studies, a consistent phenotype emerges: vagal reactions are common during PVI, whereas durable autonomic denervation surrogates—resting heart rate (HR) increase and heart rate variability (HRV) suppression—are smaller than with thermal energy;^133–137^

A key challenge in the available literature is that ‘autonomic effects’ are frequently treated as a single construct. Here, three related but non-equivalent outputs are separated:

1. Acute vagal reactions during energy delivery [bradycardia, asystole, atrioventricular (AV) block], which can reflect stimulation/reflex activation or transient stunning;^134^
2. Durable intrinsic cardiac autonomic nervous system (ICANS) injury/denervation, typically inferred indirectly via resting HR/HRV and/or denervation-associated biomarkers;^135^ and
3. Targeted ganglionated plexus (GP) modulation/ablation efficacy, best supported by physiological endpoints [e.g. abolition of high-frequency stimulation (HFS) responses, changes in effective refractory period [ERP] or inducibility] and histology when available.^135,138,139^

It is important to highlight that PFA is not a uniform intervention, as factors such as waveform, catheter design, pulse number, and tissue contact can significantly influence its biological effects. Comparative statements between PFA and thermal energy should be obtained, when possible, in randomized data (e.g. ADVENT autonomic subanalysis) or direct PFA-vs.-thermal biomarker comparisons.^135,139^

##### Acute vagal responses during PFA application

In contemporary clinical practice, acute vagal reactions during PFA are frequent, sometimes dramatic, and generally manageable. Acutely, vagal reactions typically occur immediately during or shortly after energy delivery and are usually self-limited. Several constraints limit interpretation of the vagal responses produced by PFA across studies: non-uniform definitions of ‘vagal response,’ heterogeneity in anaesthesia, analgesia, and anticholinergic use, and reliance on surrogate autonomic markers rather than direct neural histology in humans.

For example, one of the biggest multicentre prospective studies using the pentaspline catheter predefined clinically relevant vagal responses as sinus bradycardia <40 bpm, asystole >6 s, AV block, or the need for temporary backup pacing. This registry reported intraprocedural vagal reactions in 27.1% of cases overall. Importantly, in the absence of anticholinergic prophylaxis, reactions occurred in 62.5% of patients, while prophylactic administration markedly reduced event rates: glycopyrrolate (0.2 mg IV) lowered reactions to 7.5%, while atropine (1 mg IV) reduced reactions to 11.3%. Glycopyrrolate was associated with fewer drug-overdose-related adverse events, whereas atropine was more frequently linked to adverse effects, including drug-induced AF.^134^

###### Mechanistic interpretation

Current evidence favours acute parasympathetic ganglion activation or stimulation, possibly followed by transient post-stimulation refractoriness, rather than permanent vagal denervation as the primary mechanism underlying these reactions. Similar to other nerve structures such as the phrenic nerve, electrical stimulation during PFA deliveries can occur on the surrounding vagal system without associated tissue damage. Supporting this interpretation, biomarker studies comparing PFA with cryoballoon ablation demonstrated substantially lower S100 protein release with PFA indicating less neural damage.^139^ In addition, a pilot clinical study (*n* = 18) reported no persistent changes in autonomic tone or nerve injury biomarkers ∼30 days after PFA-based PVI, consistent with limited durable autonomic injury in at least some patient cohorts.^140^

Some case reports have illustrated that prolonged pauses may occur after PFA despite anticholinergic treatment. One report described more than 48 s of atrial asystole following an RSPV application, with subsequent spontaneous recovery. The authors attributed this to transient vagal stimulation near right-sided vagal pathways and emphasized the need to better delineate sinoatrial and AV nodal vulnerability during PFA.^141^ Reversible electroporation could also explain some of these prolonged observations.

###### Clinical implications

*Table 2* summarizes the main findings from clinical studies reporting vagal reactions. The available evidence supports (i) routine readiness for temporary backup pacing and consideration of anticholinergic prophylaxis, favouring glycopyrrolate when appropriate; (ii) procedural strategies that prioritize RSPV isolation to reduce pause burden because vagal events’ frequency depends strongly on lesion sequencing and PV location, consistent with anatomically clustered parasympathetic inputs^133^; and (iii) heightened caution in patients with baseline nodal disease or in those susceptible to atropine-related proarrhythmia.

**Table 2:** Summary of the main findings from clinical studies reporting vagal reactions.

Study	Study type/model	Sample size	PFA system and parameters reported	Endpoints	Main findings
Giampaolo Vetta *et al.* 2025^136^	Clinical (multicentre prospective)	240 patients	Pentaspline PFA catheter; prophylactic glycopyrrolate vs. atropine vs. none	Incidence of clinically relevant vagal reactions; drug adverse events	Vagal reactions in 27.1% overall; without prophylaxis: 62.5% (asystole 33.8%, pacing 23.8%); prophylaxis reduced reactions to 7.5–11.3%; atropine associated with more drug-related adverse events and drug-induced AF
Alvise Del Monte *et al.* 2024^135^	Clinical (prospective cohort)	80 patients	Pentaspline PFA catheter. PFA-PVI with extracardiac vagal stimulation testing; left superior pulmonary vein (LSPV)-first vs. right superior pulmonary vein (RSPV)-first sequencing	Vagal response incidence; pacing requirement; acute HR change	Vagal responses in 78% with LSPV-first vs. 13% with RSPV-first; pacing 35% vs. 8%; no residual HR or extracardiac vagal stimulation (ECVS) change at procedure end
Christopher Kowalewski *et al.* 2024^146^	Clinical (mechanistic; preprint)	24 patients	Pentaspline PFA catheter. transjugular vagal stimulation (TJVS) before/after each PV; PV order randomized	TJVS-induced sinus pause and AV block duration; pause frequency by PV order	RSPV isolation produced the largest reduction in TJVS-induced pauses and AV block; LSPV applications associated with more pauses; findings support a parasympathetic mechanism involving right-sided GP regions
Anna-Sophie Eberl *et al.* 2023^143^	Clinical (case report)	1 patient	Pentaspline PFA (RSPV application); atropine administered	Rhythm effects during PFA	Prolonged but reversible atrial asystole (>48 s) during PFA; favoured transient vagal stimulation near right vagal pathways

PFA, pulsed field ablation.

##### Durable effects of PFA on ganglionated plexi and the intrinsic cardiac autonomic nervous system

The ICANS is organized around epicardial fat pads containing ganglionated plexi (GP), which integrate parasympathetic and sympathetic signalling and can facilitate AF initiation and maintenance. In thermal ablation (radiofrequency or cryoballoon), GP modulation or denervation may have an ancillary effect of PVI that may contribute to symptomatic improvement and possibly rhythm outcomes,^135^ while other studies failed to show any improvement.^143^

###### Evidence

*Table 3* provides a summary of the main studies addressing the effects of PFA in the ICANS. The most direct human evidence that routine PVI with PFA causes limited durable GP ablation comes from a mixed retrospective–prospective study that physiologically mapped GP sites using HFS and then re-tested them after PFA-PVI. Resting HR increased at 3 months after radiofrequency and cryoballoon ablation but was essentially unchanged after PFA, implying less sustained autonomic denervation with PFA.^137,138^ In the same work a detailed substudy suggested that most GPs remained functional even when they were likely within or adjacent to the ablation footprint.^138^

**Table 3:** Summary of the main studies addressing the effects of PFA in the intrinsic cardiac autonomic nervous system.

Study	Study type/Model	Sample size	PFA system and parameters reported	Endpoints	Main findings
Daniel R. Musikantow *et al.* 2023^140^	Clinical (retrospective + prospective physiological mapping)	Retrospective: 120 (40 per energy); Prospective PFA: 20	Pentaspline PFA; HFS-defined GP mapping pre/post;	HR change at 3 months; HFS vagal response persistence; GP site involvement	HR rose after radiofrequency (RF) and cryo but not PFA; post-PFA HFS reproduced vagal effects at 76% of tested GP sites, supporting minimal durable GP ablation with PFA-PVI
Shota Tohoku *et al.* 2023^141^	Clinical comparison (PFA vs. cryoballoon)	97 (PFA 54; cryo 43)	Pentaspline PFA Serum S100 measurements pre/post; cerebral MRI to address CNS confounding	Denervation-relevant biomarker (ΔS100); relation to vagal response	S100 rose in both but markedly lower with PFA; vagal responses during PFA not associated with S100 release; supports less nervous tissue destruction with PFA vs. cryo
Naoto Otsuka *et al.* 2024^147,149^	Preclinical-experimental (swine; endocardial PFA for GP)	7 animals	Non-commercial bipolar PFA at GP sites; 1500 V/400 ms or 1250 V/300 ms; 50 pulses; anatomy + fractionated potentials used to localize sites	HR-drop response to vagal stimulation; AV nodal ERP	Partial vagal response improvement (subset); stronger effects with more sites/lesions; suggested threshold of ≥3 sites and ∼10 lesions across both atria for adequate reduction
Julie Norup Hertel *et al.* 2025^144^	Preclinical-experimental (open-chest pig; epicardial GP targeting)	PFA 6; sham 2	Non-commercial saline-irrigated bipolar epicardial PFA guided by HFS response	aERP, AF inducibility/duration, histology (S100-protein in GP)	Increased aERP, reduced AF inducibility/duration; decreased GP S100-protein intensity (acute GP damage) with sparing of adjacent arteries/sympathetic network and no local electrogram amplitude loss
NEURAL AF trial, 2025^150^	Clinical (during CABG; epicardial GP ablation)	24 patients	Non-commercial Epicardial PFA system. GP ablation during surgery;	Change in AERP; HR/HRV; safety	Feasible and safe; ∼20% AERP prolongation; HR increased; no significant HRV changes at 1 month; needs randomized validation
ADVENT autonomic subanalysis, 2024^137^	Clinical (randomized trial subanalysis)	379 patients	Pentaspline PFA vs. thermal (RF or cryo); HR from ECG; HRV from 72 h Holter at 6/12 months	Change in HR and HRV at 6/12 months	Thermal ablation produced larger HR increases and greater HRV reduction than PFA; RF and cryo similar within the thermal arm

GP, ganglionated plexi, HFS, high-frequency stimulation; HR, heart rate; HRV, heart rate variability; PFA, pulsed field ablation.

Additionally, another study that compared the effects on nerves of PFA vs. cryoballon, demonstrated that neural damage biomarker S100 increased in both groups but was substantially lower with PFA. Importantly, vagal responses during PFA were not linked to higher S100 release, supporting the concept that acute pauses are not simply a marker of ‘more denervation’.^139^

Finally, the ADVENT autonomic subanalysis provided a higher-level evidence (randomized dataset) that durable autonomic denervation was attenuated with PFA compared with thermal ablation: HR increase and HRV suppression were greater in the radiofrequency/cryoballoon arms than in the PFA arm at 6 and 12 months. Taken together, these data support the conclusion that PFA translates into less chronic autonomic perturbation, despite frequent acute vagal reactions.^135^

###### Mechanisms

There are several potential mechanisms explaining reduced effects of PFA on GP. The two main constraints are geometry (distance) and tissue threshold. GPs are epicardial and often embedded in fat. Endocardial PVI lesions (even when ‘wide antral’) may not generate sufficient electric field strength at GP depth to exceed neural electroporation thresholds, even if adjacent myocardium is ablated at lower thresholds. This was observed in early preclinical work, which noted that endocardial PFA had limited impact on epicardial GPs owing to distance, whereas epicardial delivery might be effective.^146^ Also, a potential mechanism is neural regeneration after electroporation injury suggesting that even when neural elements are affected, durable denervation may not be guaranteed.^148^

GP-related effects time course can be framed as follows:

- Early post-procedure (hours–days): transient modulation or stunning may occur (e.g. sequence effects reducing subsequent vagal response burden), but durable ‘denervation endpoints’ are usually not met in endocardial PVI practice.^133^
- Delayed (months): compared with thermal ablation, autonomic profile changes (HR increase, HRV reduction) are smaller after PFA, implying less durable autonomic remodelling/denervation in typical PVI workflows.^135^

###### Feasibility in a clinical setting

Given the above mechanistic explanation, targeted GP ablation with PFA in a clinical setting becomes more plausible in two scenarios:

- Endocardial PFA with dedicated GP-focused dosing and strategy. Preclinical data provide early signals that endocardial PFA can partly blunt vagal reflexes and modify nodal electrophysiology, but results are variable and appear to depend on the extent and number of application sites. In a porcine model, investigators defined a positive vagal response as >15% HR drop and delivered bipolar PFA at GP sites; vagal responses improved in 3/7 (trend) and AV nodal ERP improved, but complete elimination was not achieved.^144^ Increasing the number of sites and lesions correlated with greater reduction in HR drop; the authors concluded that at least 3 sites and 10 lesions across both atria were needed for an ‘adequate’ vagal response decrease.^145^
- Epicardial PFA directly applied to GP sites. This setting provides the most convincing mechanistic evidence. In an open-chest pig model targeting five epicardial GP sites, epicardial bipolar PFA increased atrial ERP, reduced AF inducibility (100% → 33%) and AF duration, and demonstrated decreased S100 protein intensity within GP cytoplasm/membrane (interpreted as acute GP damage) while sparing surrounding arteries and sympathetic network.^146^ That pattern, functional autonomic modification without obvious atrial myocardial electrogram loss, suggests that epicardial PFA could be used for ‘cardioneuroablation’.

The NEURAL AF study (single-arm prospective during CABG) showed feasibility and acute physiologic effect of PFA in GPs: 24 patients underwent epicardial GP ablation with pulsed-field energy, yielding ∼20% atrial ERP prolongation and a rise in mean HR, without significant HRV change at 1 month.^147^

However, several constraints are particularly relevant for assessing the long-term effects of PFA on the GP:

- Endpoint ambiguity: abolition of an HFS response, resting HR increase, and biomarker release are related but non-equivalent endpoints; they can diverge.^138^
- Access and anatomy confounding: endocardial vs. epicardial delivery fundamentally changes electric field distribution; open-chest pig models may overestimate feasibility relative to percutaneous human epicardial access constraints.^146^
- Dose heterogeneity: ‘PFA’ is not a single dose; waveforms, electrode geometry, pulse counts, and field strength vary across systems, limiting generalization.^144,145^

To conclude, PFA-PVI should not be expected to confer the same ‘bonus denervation’ often attributed to thermal ablation. If a clinical hypothesis relies on GP ablation (e.g. vagal AF or vasovagal syncope-oriented cardioneural ablation), current evidence suggests that standard endocardial PFA-PVI is unlikely to be sufficient, and that dedicated strategies, potentially epicardial, are required and remain investigational.^138^ Conversely, attenuated durable denervation may be beneficial by reducing unwanted long-term vagal impairment syndromes, but this remains an inference requiring prospective testing.^135^

## Part III: PFA effects on the conduction system

### Effect of PFA on the conduction system

The effects of PFA on the conduction system, and specifically on the atrioventricular node (AVN) and AV conduction properties are nowadays still a topic of active research as many mechanisms remain unclear. When considering conduction system effects of PFA, it is essential to separate phenomena that may appear similar on surface ECG but have distinct mechanisms and implications:

- Vagally mediated nodal block (functional, common during PVI, usually preventable and caused by transient reversible electroporation) or stunning of conduction system structures (often reversible and dose-dependent).^149^
- A rare and yet to be proved potential mechanism related to ischaemia-mediated conduction disturbance from proximity-related coronary spasm during non-PV PFA (rare, but potentially severe).^130,136^
- Irreversible electroporation leading to permanent ablation of conduction system structures.

#### Transient observations

Most reports using PFA near different structures of the conduction system demonstrate the possibility of triggering transient conduction disturbances. Effects observed can greatly vary from study to study. For example, a recent clinical study proposed that PFA delivery close to the AVN for the treatment of atrioventricular nodal re-entrant tachycardia (AVNRT) was safe,^150^ while other studies showed AV conduction delay and transient AV block.^151–153^

Other case reports include prolonged atrial asystole during PFA-PVI after an RSPV application, with sinus node recovery only after >48 s; despite atropine, recurrence occurred.^141^ Additionally, transient sinoatrial node dysfunction was observed shortly after PFA-PVI, resolving within 6 h; direct stimulation during ablation and reversible electroporation stunning were discussed as plausible mechanisms.^154^ His–Purkinje conduction disturbances during cavotricuspid isthmus PFA led to right bundle branch block plus left posterior fascicular hemiblock plus PR prolongation, resolving within 12 h.^155^

The available evidence suggests that while PFA in standard PVI workflows is often delivered at a distance from compact AV nodal tissue, conduction tissue can be affected when fields reach nodal/His–Purkinje regions (or when autonomic reflexes predominate).^155^

Preclinical models have directly interrogated conduction tissue vulnerability to PFA. A preclinical canine study showed His bundle voltage-dependent responses. When applying pulsed electric fields at the site of maximal His potential all animals developed transient third-degree AV block, with recovery time increasing in a voltage-dependent fashion (40 s at 600 V up to 438 s at 1800 V).^149^ AH intervals prolonged, HV did not significantly change, and Masson staining showed no apparent structural damage to His bundle cells. This pattern is most consistent with reversible conduction block due to transient functional impairment (reversible electroporation, oedema, or injury to adjacent myocardium supporting conduction continuity), rather than necrosis of specialized conduction fibres.^149^ Another chronic canine model performed ablation using bipolar nanosecond electroporation across the basal interventricular septum, transient infrahisian AV block could be induced in all animals with gradual recovery; right bundle branch block could occur transiently, and more extensive dosing could yield longer-lasting conduction block (including persistent right bundle branch block morphology).^156^ Finally, another study demonstrated that pulsed electric fields can acutely modulate Purkinje potentials with relative resistance of the His bundle in certain experiments (e.g. disappearance of smaller Purkinje potentials with preserved His signals), suggesting regional differences in susceptibility and/or local field distribution at catheter positions.^157,158^

#### Intentional permanent ablation

It is important to highlight that although PFA may display myocardial tissue preferential ablation, studies have demonstrated that the conduction system is not always spared. PFA has also been demonstrated as a feasible option for AV node ablation. Some studies have demonstrated that conduction tissue can be ablated by PFA under specific catheter positioning and dosing.^151,159,160^ In a preclinical model, authors demonstrated that that monopolar focal PFA delivery directly at the AV junction resulted in sustained AV block. In contrast, PFA delivery indirectly at the AV junction from within the non-coronary cusp resulted only in transient AV conduction delay, highlighting the importance of distance to the AV node. The same study also demonstrated PFA ability to permanently ablate AV node with focal PFA.^151^

#### Mechanisms

- *Field distribution:* PFA effects on the conduction system are largely dependent on electric field dosing, which may be controlled by distance to the ablation catheter and applications repetition and overlap. Additionally, the intrinsic fibrotic structure of the conduction system with a dense network of fibrosis surrounding the specialized conduction cells may create some insulating effects creating heterogeneous field distributions leading to reversible block of specialized cells.^149^
- *Reversible electroporation and oedema:* Preclinical models suggest that these two mechanisms could also partially explain the effects observed. For example, the voltage-dependent recovery relationship observed in preclinical models targeting His fibres supports graded reversible impairment rather than a binary effect supporting reversible electroporation mechanisms.^149^ Additionally, biophysics of electroporation predict that cells with a smaller diameter, as the AVN cells, may be less susceptible to similar electric field exposure, leading to reversible electroporation rather than irreversible injury. Oedema observed in the PFA could also partially explain transient effects beyond the reversible electroporation recovery time frame.
- *Other mechanisms* can cause conduction deterioration: although not yet completely understood, some authors have speculated with the hypothesis that coronary spasm could cause transient ischaemia and trigger AV block or ventricular arrhythmias during PFA at locations such as the cavotricuspid isthmus (CTI), where coronary anatomy is relevant.^130^

#### Clinical implications

Practical considerations regarding conduction system effects to consider when using PFA include:

*For PFA-PVI workflows:* Anticipate nodal pauses (backup pacing/anticholinergic strategy), interpret many AV blocks as functional vagal phenomena, and monitor higher-risk patients more closely.^134^

*For non-PV applications (CTI, septal targets):* Recognize that PFA can affect conduction tissue and can be associated with coronary spasm-related ischaemia; consider cautious dosing, catheter positioning, and, until better evidence exists, energy-source selection based on substrate location and operator experience.^130,136,149^

Future research should focus on controlled human studies to quantify safety margins and define the feasibility of reversible ‘electroporation mapping’ protocols around His/AV node regions.

## Data Availability

The data underlying this article will be shared on reasonable request to the corresponding author.
